# The clinical and prognostic correlation of HRNPM and SLC1A5 in pathogenesis and prognosis in epithelial ovarian cancer

**DOI:** 10.1371/journal.pone.0179363

**Published:** 2017-06-13

**Authors:** Kathrine Bjersand, Tomas Seidal, Inger Sundström-Poromaa, Helena Åkerud, Ingiridur Skirnisdottir

**Affiliations:** 1 Department of Women’s and Children’s Health, Uppsala University, Uppsala, Sweden; 2 Department of Pathology, Halmstad Medical Center Hospital, Halmstad, Sweden; 3 Department of Immunology, Genetics and Pathology, Uppsala, Sweden; University of South Alabama Mitchell Cancer Institute, UNITED STATES

## Abstract

**Objectives:**

To evaluate the prognostic effect of the Heterogeneous nuclear ribonucleoprotein type M (HNRPM) and Solute carrier 1A5 (SLC1A5) in FIGO-stages I-II epithelial ovarian cancer.

**Methods:**

A retrospective cohort study was designed to investigate the prognostic effect of HNRPM and SLC1A5, and the association with clinical-pathologic characteristics in 131 patients with FIGO-stages I-II epithelial ovarian cancer. Tissue microarrays were constructed and protein levels were assessed by immunohistochemistry (IHC).

**Results:**

Positive HRNPM status was associated with positive staining for PUMA (P = 0.04), concomitant PUMA and p21 staining (P = 0.005), and VEGF-R2 (P = 0.003). Positive SLC1A5 staining was associated with positive staining of p27 (P = 0.030), PUMA (P = 0.039), concomitant PUMA and p27 staining, and VEGF-R2 (P = 0.039). In non-serous tumors (n = 72), the SLC1A5 positivity was associated with recurrent disease (P = 0.01). In a multivariable logistic regression analysis FIGO-stage (OR = 12.4), tumor grade (OR = 5.1) and SLC1A5 positivity (OR = 0.1) were independent predictive factors for recurrent disease. Disease-free survival (DFS) in women with SLC1A5-positive non-serous tumors was 92% compared with of 66% in patients with SLC1A5-negative non-serous tumors (Log-rank = 15.343; P = 0.008). In Cox analysis with DFS as endpoint, FIGO-stage (HR = 4.5) and SLC1A5 status (HR = 0.3) were prognostic factors.

**Conclusions:**

As the proteins HRNPM and SLC1A5 are associated with the cell cycle regulators p21 or p27, the apoptosis regulators PTEN and PUMA, and the VEGF-R2 it is concluded that both proteins have role in the pathogenesis of ovarian cancer. In patients with non-serous ovarian cancer SLC1A5 protects from recurrent disease, presumably by means of biological mechanisms that are unrelated to cytotoxic drug sensitivity.

## Introduction

Epithelial ovarian cancer is associated with diffuse symptoms, diagnosis in advanced stage and is the most common cause of mortality among women with gynecological malignancies. About 30% of patients present in stage I-II, with tumor spread limited to the pelvis. Golden standard is surgical treatment, which includes optimal staging and extensive cyto-reductive surgery in more advanced cases. Surgery is usually followed by adjuvant platinum and taxane-based chemotherapy [1]. As clinical-pathological parameters are insufficient for prediction of prognosis as well as response to chemotherapy, additional methods are needed to individualize treatment.

DNA is constantly being damaged due to errors in replication and external factors, such as ionizing radiation. Left unrepaired, this results in unstable chromosomes and mutations that might drive cancer development. Cell cycle control is a central process for prevention of cancer, by inducing cell cycle arrest and apoptosis in damaged tissue [2]. In a previous study from our group it was shown that loss of a small region in chromosome 19q was associated with recurrent disease in women with serous ovarian cancer [3]. Heterogeneous nuclear ribonucleoprotein type M (HNRPM) and Solute carrier 1A5 (SLC1A5) were among the genes of interest found in this region of chromosome 19q. Furthermore, we have previously shown that Napsin A was more frequently expressed in clear cell carcinoma than in other histopathological subtypes of epithelial ovarian cancer [4].

Heterogeneous nuclear ribonucleoproteins (HNRPs) are mRNA binding proteins, all present in the nucleus, but some with the ability to move between the nucleus and the cytoplasm. HNRPs have central roles in cell signaling, chromosome stability and cell cycle regulation and, by way of these functions, this protein family has the potential to regulate tumor development [5]. For instance, in breast cancer cells HNRPM has been shown to participate in processes that lead to epithelial–mesenchymal transition and metastatic disease [6,7]. Most evidence supports that HNRP is an intracellular protein, but Languinge et al. [8] suggests that HRNPM acts as a membrane receptor for cancer embryonic antigen (CEA), a marker that often correlates with metastatic spread of colorectal cancer. HRNPM seems to be dependent upon binding to CEA for signaling, but its roll in colorectal cancer metastatic disease is not fully understood.

Solute carriers (SLC) is a group of membrane influx transporters with a diverse range of substrates such as sugars, amino acids, vitamins, nucleotides, inorganic ions, trace minerals and drugs. As a result of altered metabolism of these substrates in cancer cells, SLCs gene expression may be modified. Some SLCs may be up-regulated to meet the increased energy demand of the growing tumor; thus blocking them may limit the transport of energy and tumor growth. SLCs also mediate the cellular uptake of hydrophilic drugs and could be useful tools for drug delivery to cancer cells. However, on the other hand, SLC genes may be up- or down regulated in the development of drug resistance [9]. During tumor progression tumor cells overcome the allowed number of normal cell doubling, and SLC1A5 is known to be a part of this process [10].

Thus, given the fact that loss of a small region in chromosome 19q is associated with increased risk of recurrent disease, the aim of this study was to investigate the impact of the proteins HRNPM and SLC1A5, both of which are found in this region, on clinical factors, pathogenesis and prognosis in ovarian cancer. Furthermore we evaluated the relationship between these two proteins and the cell cycle regulators p21 and p27, the apoptosis regulators PTEN and PUMA and the angiogenesis regulator VEGF-R2 in patients with FIGO I-II stage ovarian carcinoma.

## Material and methods

### Ethics

All samples were collected with the patients’ informed consent, in compliance with the Helsinki Declaration [11], and used in accordance with the Swedish Biobank Legislation and Ethical Review Act, approved by the Uppsala Ethical Review Board, decision (ref.UPS-03-477).

### Patients and tissue material

In total, 123 patients out of 131 patients with FIGO-stage (1988 FIGO-staging) I-II epithelial ovarian cancer, who underwent primary surgery and post-surgical chemotherapy in the Uppsala-Örebro Medical Region during the 5-year period from January 1, 2000 to December 31, 2004, were included in this study. The primary surgery was performed at nine different surgical gynecological departments and the staging procedure was done at the time of primary surgery. Modified surgical staging according to the EORTC surgical staging [12] was undertaken in 34 (28%) of the 123 cases, and in the remaining 89 (72%) patients surgical staging was regarded as minimal or inadequate according the same guidelines.

Patients’ characteristics, including age, BMI, performance status of the patients (WHO), FIGO-stage, serous/non-serous histology and types of ovarian tumors (Type I and Type II), according to combinations of histological subtype and FIGO-grade, are summarized in Table 1. All patients had chemotherapy 4–6 weeks after the primary surgery, most commonly paclitaxel 175 mg/m^2^ and carboplatin (AUC = 5) at 3-week intervals usually for four courses (n = 98), or single-drug carboplatin in 4–6 courses (n = 25). The mean follow-up time was 65 months (range 5–110 months). Survival was defined as date of confirmed histological diagnosis after primary surgery to date of recurrence, death, or last visit.

**Table 1:** Patients’ characteristics (n = 123). Data given as median (range) or n (%).

Age (median)	59.0 (range 25–84)
BMI ≤ 25 kg/m^2^	66 (55.0)
BMI > 25 kg/m^2^	54 (45.0)
WHO performance status	
0	34 (27.6)
1	61 (50.4)
2	21 (17.1)
3	6 (4.9)
FIGO stage	
IA	37 (30.1)
IB	5 (4.1)
IC	61 (49.6)
II	20 (16.2)
Histopathology^a^	
Serous ovarian tumors	49 (40.5)
Non-serous ovarian tumors	72 (59.5)
Mucinous	19 (26.4)
Endometrioid	40 (55.6)
Clear cell	13 (18.0)
Types of ovarian tumors	
Type I tumors^b^	71 (58.1)
Low-grade (G1) serous	12
Mucinous (G1 + G2 + G3)	19
Low-grade endometroid (G1 + G2)	27
Clear cell	13
Type II tumors	52 (41.9)
High-grade serous (G2 + G3)	37
High-grade endometroid (G3)	13
Anaplastic	2

^a^ The two tumors of anaplastic histology were excluded

^b^ Tumors grouped in Type I and Type II tumors according to combination of histological subtype and FIGO-grade.

In our previous study on serous ovarian cancer in FIGO-stages I-II, DNA was extracted from formalin-fixed samples containing tumor cells from ovarian tumors [3]. Tumor samples from 37 patients were analyzed for allele-specific copy numbers using OncoScan single nucleotide polymorphism arrays from Affymetrix and the bioinformatics tool Tumor Aberration Prediction (TAPS). Genomic gains, losses, and loss-of heterozygosity (LOH) associated with recurrent disease were identified in the regions where the greatest difference in LOH were seen, on chromosome 19q. In this region, 56 genes were described as potential candidate genes associated with recurrent disease. Among these, nine genes had previously been described to be expressed in ovarian carcinoma according to the Human Protein Atlas; both HRNPM and SLC1A5 were among these.

### Sampling and tissue microarray construction of ovarian cancer tissue

Paraffin-embedded tumor tissue from the primary surgery was used. Following staining with hematoxylin and eosin, the tumors were classified and graded by a single pathologist. The tissue microarrays were constructed as described previously [13]. In brief, tumor tissues were embedded in paraffin and 5 μm sections stained with hematoxylin-eosin were obtained to select representative areas for biopsies. Core tissue biopsy specimens (diameter 0.6 mm) were taken from these areas of individual donor paraffin blocks and precisely arrayed into a new recipient paraffin block with a custom-built instrument. Two tissue core specimens (diameter 0.6 mm) from all 131 ovarian carcinomas were arranged in three recipient paraffin blocks. The presence of tumor tissue on the arrayed samples was verified by hematoxylin-eosin-stained sections by a pathologist. The tissue microarray construction was done at the Department of Pathology, the University Hospital MAS in Malmo in South-Sweden.

### Immuno-histochemistry and interpretation

**Fig 1:**
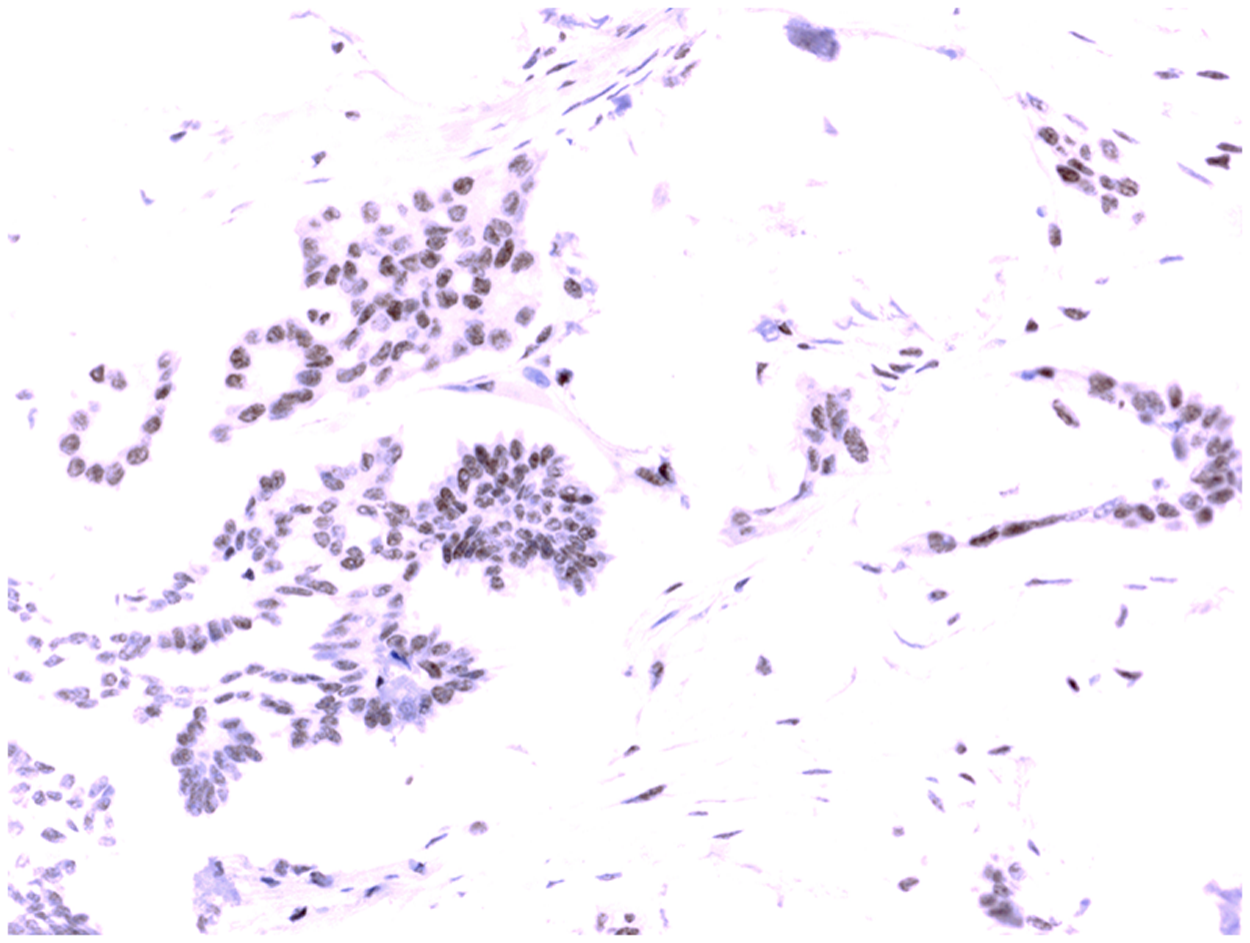
Serous ovarian carcinoma with nuclear positivity for HRNPM (brown staining).

**Fig 2:**
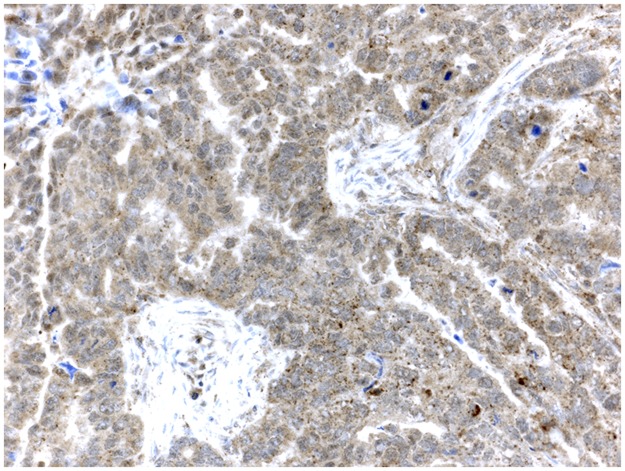
Endometrioid ovarian carcinoma, strongly positive for SLC1A5 (brown staining).

Five μm thick sections were cut from each multi-tissue block and were put on coated slides and dried overnight at 37°C. The sections were pre-treated by heath-induced epitope retrieval in target-retrieval solution (Dako, Glostrup, Denmark), pH = 6 or EDTA buffer pH = 9, for 7 + 7 minutes in microwave oven (99°C). Blocking with peroxidase was performed for 5 minutes. The slides were counterstained with hematoxylin for 2 minutes. The following monoclonal primary antibodies were used: HNRNPM, LS-B4384, LifeSpan BioScience, Mouse monoclonal, 1:50 and SLC1A5, LS-A9042, LifeSpan BioScience, Rabbit polyclonal, 1:150. Information about the primary antibodies and immuno-histochemical analyses can be found in previous studies [14–18]. The immunostainings were performed in an Autostainer automated machine (Dako) using the REAL Envision detection system (Dako). The IHC analyses and interpretation were performed at the Department of Pathology, Halmstad Medical Central Hospital. The IHC stains were interpreted by the authors IS and TS. At the time for evaluation, no information was available on the specific diagnosis and prognosis for the individual cases. Of the 131 available tumor samples, staining for HRNPM was successful in 123 tumor samples and corresponding number for SLC1A5 was 121 tumor samples. A semi-quantitative analysis [19] was used and the stainings were graded as negative, +, ++, and +++ for p21, p27, PTEN, VEGF-R2 and SLC1A5. All those markers were dichotomized into negative and positive (+, ++, +++) cases [20]. However, for both PUMA and HRNPM, weak positive staining (+) was graded as negative (-) and moderate /strong staining (++ / +++) graded as positive. The staining for p21 was considered to be positive when there was a strong, granular staining of the nuclei in the majority of tumor cells; the stainings for p27 and PTEN were considered to be positive when strong granular staining of the nuclei and cytoplasm of the tumor cells was found. Staining for VEGF-R2 was both membranous and cytoplasmic. The localization for HRNPM staining was distinctly nuclear with a weak cytoplasmic background in the tumor cells. As negative staining of the cytoplasm hardly was detected in this series of patients, our findings were limited to weak or moderate / strong staining of cytoplasm (Fig 1). Positive staining for SLC1A5 was detected as diffuse staining of the cytoplasm with a very strong positivity of large globuli in the cytoplasm considered as Golgi-positivity (Fig 2). For PUMA, negative staining of cytoplasm in tumor cells could not be detected in this series of patients. Therefore, findings were limited to weak or moderate / strong staining of the cytoplasm.

### Statistical analyses

The Pearson’s Chi-square test was used for testing proportional differences in univariate analyses. The survival curve was generated using the Kaplan-Meier technique and differences between these curves were tested by the log-rank test. The logistic regression model was used for both univariate and multivariate analyses with recurrent disease as the end point. Furthermore, the univariate and multivariate Cox regression model was used with disease-free survival (DFS) as the endpoint. All tests were two-sided and the level of statistical significance was p ≤ 0.05. The STATISTICA 13.0 (StatSoft ^™^) statistical package was used for the analyses.

## Results

### Patients

Patients’ characteristics are summarized in Table 1. The study population included 71 type I tumors (58.1%) and 52 type II tumors (41.9%). The majority (83.8%) of the patients had a stage I disease and the majority (58%) of the tumors were classified as Type I tumors. Primary cure was achieved in all 123 patients. The total number of recurrences in the complete series was 32 out of 123 (26%), and 22 of these patients (67.8%) died due to the disease during the follow-up. In the complete series, recurrent disease was significantly associated with FIGO sub-stages (IA-IB/IC/II) (P< 0.0002), FIGO-grade (P = 0.023), residual disease (P = 0.001), and Type of tumor (I/II) (P = 0.023). In the complete series the 5-year disease-free survival rate was 68%, the disease-specific survival rate 76%, and the overall survival rate 71%

### Immunohistochemistry; Expression of HRNPM and SLC1A5 proteins and relation to clinical-pathological factors and survival

HRNPM was only found in the nucleus of tumor cells, and positivity for HRNPM was detected in 85 (61%) out of the 123 tumors (Fig 1). HRNPM status was not associated with serous / non-serous tumors, FIGO-stage, type of tumors, or recurrent disease (Table 2). High grade (G2) tumors were to a higher extent HRNPM-negative, although the difference was not significant (P = 0.066).

**Table 2:** Status of protein expression in tumors of the HRNPM (N = 123) and SLC1A5 (N = 121) vs. clinical and pathological features. Data expressed as n (%).

Expression	HRNPM+	HRNPM-	P-value	SLC1A5+	SLC1A5-	P-value
	85 (61)	38 (39)		92 (86)	29 (24)	
Histopathology^a^						
Serous	34 (41)	15 (39)		37 (41)	11 (38)	
Non-serous	49 (59)	23 (61)		53 (59)	18 (62)	
			0.876			0.761
Tumor grade						
G1	22 (25)	5 (13)		21 (23)	6 (21)	
G2	25 (30)	19 (50)		34 (37)	9 (31)	
G3	38 (45)	14 (37)		37 (40)	14 (48)	
			0.066			0.715
Type of tumors						
Type I	47 (55)	24 (63)		51 (55)	19 (65)	
Type II	38 (45)	14 (37)		41 (45)	10 (35)	
			0.414			0.337
FIGO stage						
IA-IB	31 (37)	11 (29)		34 (37)	7 (24)	
IC	39 (46)	22 (58)		43 (47)	18 (62)	
II	15 (17)	5 (13)		15 (16)	4 (14)	
			0.466			0.355
Recurrent disease						
Without	63 (74)	28 (74)		71 (77)	19 (65)	
With	22 (26)	10 (26)		21 (23)	10 (35)	
			0.959			0.209

^a^ The two tumors of anaplastic histology were excluded

Positive staining for SLC1A5 was detected as diffuse staining of the cytoplasm with a very strong positivity of large globuli, considered as Golgi-positivity (Fig 2). Positivity for SLC1A5 was detected in 92 (86%) out of the 121 available tumors (Table 2). The SLC1A5 status was not associated with serous / non-serous tumors, tumor grade, FIGO-stage, type of tumor, or recurrent disease (Table 2).

### HRNPM status in association with markers of the cell cycle, apoptosis and angiogenesis

The HRNPM status was not associated with presence of p21, p27 or PTEN (Table 3). However, positivity of HRNPM was more frequently found in tumors positive for PUMA (P = 0.044). Furthermore, HRNPM status was associated with concomitant PUMA and p21 status of the tumors (P = 0.005). Thus, HRNPM-positive tumors were predominantly detected in tumors positive for PUMA and negative for p21, and vice versa, negative for PUMA and positive for p21. In contrast, HRNPM-negative tumors usually were negative for both PUMA and p21. Finally, HRNPM positive tumors were more frequently found in to be positive for VEGF-R2 (P = 0.003).

**Table 3:** Protein expression of apoptosis and angiogenesis regulators versus HRNPM status in ovarian tumors (n = 123).

Protein expression	HRNPM+ n (%)	HRNPM- n (%)	P-value
	85 (61)	38 (39)	
p21+	35 (42)	9 (24)	
p21-	49 (58)	28 (76)	0.067
p27+	50 (59)	22 (58)	
p27-	35 (41)	16 (42)	0.923
PTEN+	14 (17)	11 (29)	
PTEN-	70 (83)	27 (71)	0.120
PUMA+	43 (51)	12 (32)	
PUMA-	41 (49)	26 (68)	0.044
PUMA+p21+	17 (20)	6 (16)	
PUMA+p21-	18 (22)	3 (8)	
PUMA-p21+	26 (31)	6 (16)	
PUMA-p21-	22 (27)	22 (60)	0.005
PUMA+p27+	27 (32)	8 (21)	
PUMA+p27-	21 (25)	14 (37)	
PUMA-p27+	17 (20)	3 (8)	
PUMA-p27-	19 (23)	13 (34)	0.104
VEGF-R2+	72 (85)	23 (61)	
VEGF-R2-	13 (15)	15 (39)	0.003

### SLC1A5 status and association with markers of the cell cycle, apoptosis, and angiogenesis

SLC1A5 staining was associated with p27 positivity (P = 0.030), but not to the p21 status of tumors (Table 4). Furthermore, SLC1A5 positive tumors usually displayed positivity for PTEN (P = 0.034), PUMA (P = 0.039), and concomitant p27 and PUMA positive staining (P = 0.016). Lastly, SLC1A5 positive tumors were usually also positive for VEGF-R2 (P = 0.039).

**Table 4:** Protein expression of apoptosis and angiogenesis regulators versus SLC1A5 status in ovarian tumors (n = 121).

Protein expression	SLC1A5+ n (%)	SLC1A5- n (%)	P-value
	92 (76)	29 (24)	
p21+	35 (38)	9 (32)	
p21-	56 (62)	19 (68)	0.545
p27+	59 (64)	12 (41)	
p27-	33 (36)	17 (59)	0.030
PTEN+	23 (25)	2 (7)	
PTEN-	68 (75)	27 (93)	0.034
PUMA+	45 (49)	8 (27)	
PUMA-	46 (51)	22 (73)	0.039
PUMA+p21+	21 (23)	2 (7)	
PUMA+p21-	14 (16)	7 (25)	
PUMA-p21+	24 (27)	6 (21)	
PUMA-p21-	31 (34)	13 (47)	0.172
PUMA+p27+	30 (33)	4 (14)	
PUMA+p27-	27 (30)	8 (28)	
PUMA-p27+	16 (17)	3 (10)	
PUMA-p27-	18 (20)	14 (48)	0.016
VEGF-R2+	75 (82)	18 (62)	
VEGF-R2-	17 (18)	11 (38)	0.039

### Clinical characteristics in non-serous tumors and serous tumors

Age, BMI, FIGO-stage, tumor grade and recurrent disease were compared between non-serous and serous tumors. Patients with non-serous tumors were younger than patients with serous tumors (57.1 years vs. 61.4 years, respectively, P = 0.045). No differences in BMI (P = 0.847), FIGO stages (P = 0.178), tumor grade (P = 0.529), or recurrent disease (P = 0.227) were found between the two subgroups. Furthermore, there were no differences between serous and non-serous tumors according to SLC1A5 positivity (Table 2), or the type of post-surgical chemotherapy that had been given (P = 0.607).

### Non-serous tumors

The three subgroups of non-serous tumors (mucinous, endometroid and clear cell) were compared according to status of the cell cycle regulators p21, p27, the apoptosis regulators PTEN, PUMA, the angiogenesis regulator VEGF-R2 and HRNPM and SLC1A5. According to these analyses, clear cell tumors more often stained positive for p21 and mucinous tumors more often stained negative for p21 (P = 0.033). Endometroid and clear cell tumors tended to stain positive for p27 and mucinous tumors negative for p27 (P = 0.07). No differences in PTEN, PUMA, VEGF-R2, HRNPM, or SLC1A5 status were found between three histological subgroups.

### SLC1A5 status and clinic-pathological factors in non-serous tumors

**Fig 3:**
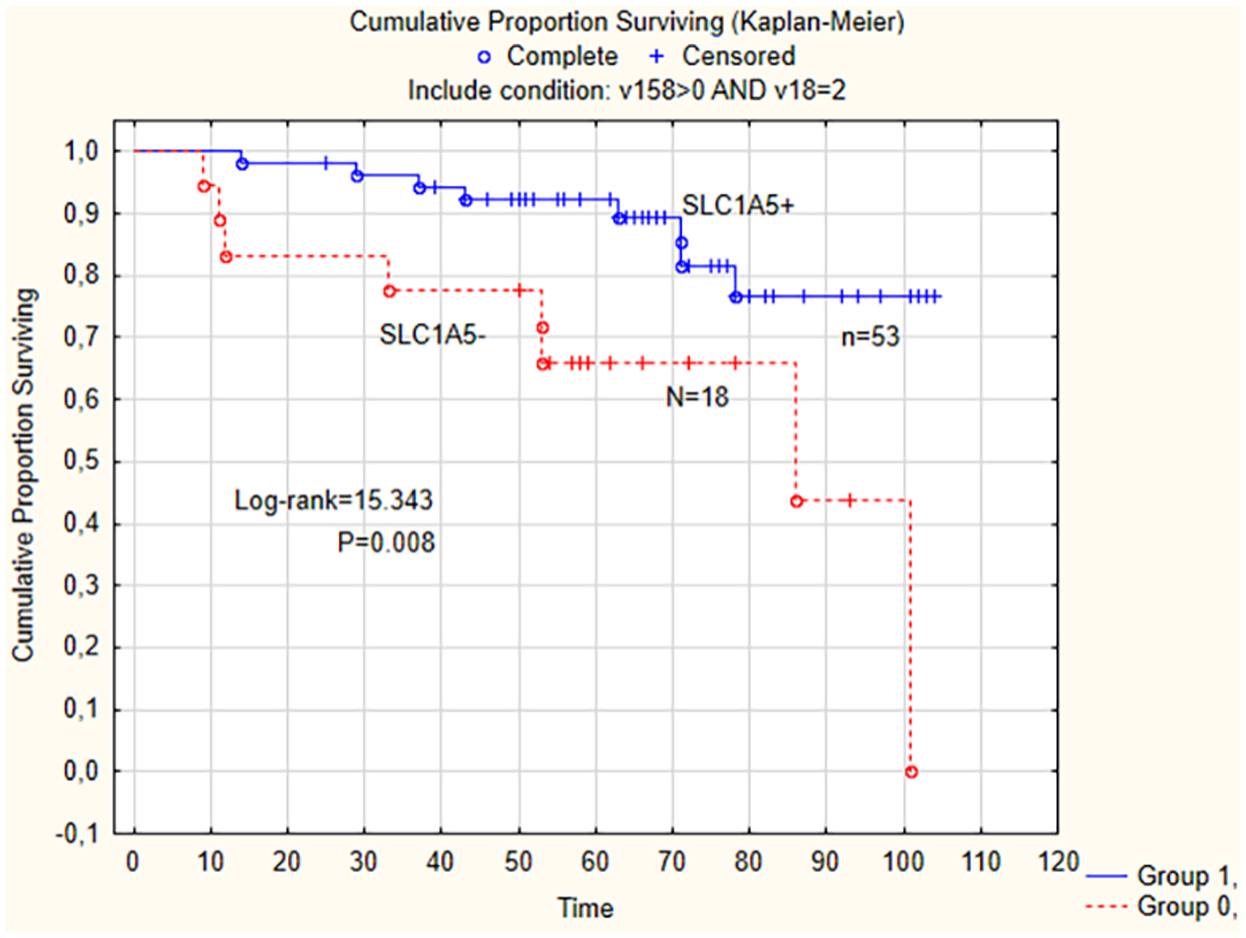
The 5-year disease-free survival for the subgroup of patients with SLC1A5 positivity of tumors was 92% compared with the 5-year DFS (disease-free survival) of 66% for the subgroup of patients with SLC1A5 negativity of tumors.

The association between SLC1A5 status in non-serous tumors and clinical- and pathological features and survival was explored in patients with information on SLC1A5 status (n = 71). No association between SLC1A5 positivity and tumor grade, FIGO-stage or type of tumors was noted. However, SLC1A5 status was significantly associated with recurrent disease (P = 0.01). Thus, among the 53 patients with SLC1A5-positive non-serous tumors only eight (15%) had recurrent disease, whereas the corresponding number in women with SLC1A5-negative tumors was 8/18 (44%). The 5-year disease-free survival in women with SLC1A5-positive non-serous tumors was 92% compared with the 5-year disease-free survival of 66% for women with SLC1A5-negative tumors (Log-rank = 15.343; P = 0.008) (Fig 3).

For the subgroup of patients with SLC1A5 positive non-serous tumors (n = 53), no difference in recurrent disease according to given treatment (paclitaxel and carboplatin versus single drug carboplatin) was found (P = 0.196). Furthermore, among the patients with non-serous tumors who received postoperative treatment with both paclitaxel and carboplatin (n = 56), recurrent disease was found in only 8 (18%) out of 45 patients with SLC1A5-positive tumors compared with 6 (55%) out of the 11 patients with SLC1A5-negative tumors (P = 0.011). Thus, the SLC1A5 status was significantly associated with recurrent disease, in a subgroup of patients, who all received the same type of chemotherapy.

### SLC1A5 status and different biomarkers in non-serous tumors

The associations between SLC1A5 status in non-serous tumors and the cell cycle regulators (p21 and p27), the apoptosis regulators (PTEN and PUMA), and the angiogenesis regulator VEGF-R2 were explored. SLC1A5 positivity was associated with the p27 positive staining of non-serous tumors (P = 0.033), where concomitant positivity of SLC1A5 and p27 was found in 33 (62%) out of the 53 tumors with positive SLC1A5 status compared with positivity for p27 in only 6 (33%) out of the 18 tumors with negative SLC1A5 status. The SLC1A5 status was also associated with the VEGF-R2 status in non-serous tumors (P = 0.009). Thus concomitant positivity for SLC1A5 and VEGF-R2 was found in 43 (81%) out of the 53 SLC1A5-positive tumors, whereas the corresponding rate was 9 (50%) in SCL1A5-negative tumors.

### Multivariable analysis

Results are shown for univariate and multivariate logistic regression analysis with recurrent disease as endpoint in Table 5. The FIGO-stage, tumor grade and SLC1A5 status were all independent predictive factors for recurrent disease. The odds ratio of 0.134 suggests that patients with SLC1A5-positive non-serous tumors had 86% reduced risk of recurrent disease. In a further multivariate logistic regression analysis with recurrent disease as endpoint in (Table 6) with Type (I/II) of tumors and staging surgery instead of tumor grade and FIGO-stage I/II, the SLC1A5 status was still an independent predictive factor for recurrent disease in patients with non-serous tumors. Furthermore, results are shown for univariate and multivariate Cox analyses with disease-free survival (DFS) as endpoint in Table 7. In this analysis, both FIGO-stage and SLC1A5 status were significant and independent prognostic factors. The value HR = 0.284 (P = 0.024) for SLC1A5 indicates that patients from that subgroup had a 71% reduced risk to die due to their ovarian cancer.

**Table 5:** Predictive factors for recurrent disease (bivariate and multivariable logistic regression) in patients with non-serous tumors (n = 72).

Variable	Bivariate analysis		Multivariable analysis		P-value
	OR	95% CI	AOR	95% CI	
Age	0.99	0.95–1.04	1.02	0.96–1.09	0.472
Stage (I/II)	10.20	2.60–40.0	12.42	2.28–67.9	0.003
Grade^a^	6.88	1.90–25.0	5.06	1.10–23.2	0.033
HRNPM positive	0.51	0.16–1.65	0.46	0.10–2.13	0.311
SLC1A5 positive	0.22	0.06–0.75	0.13	0.03–0.67	0.013

^a^ Grade (G1+G2 vs. G3)

**Table 6:** Predictive factors for recurrent disease (bivariate and multivariable logistic regression analysis) in patients with non-serous tumors.

Variable	Bivariate analysis		Multivariable analysis		P-value
	OR	95% CI	AOR	95% CI	
Age	0.99	0.95–1.04	1.01	0.96–1.06	0.808
Staging surgery^a^	0.43	0.16–1.17	0.67	0.16–2.78	0.571
Type (I/II)	2.46	1.10–5.49	5.74	1.25–26.3	0.021
HRNPM positive	0.51	0.16–1.65	0.41	0.10–1.61	0.193
SLC1A5 positive	0.22	0.06–0.75	0.23	0.06–0.89	0.029

^a^ Staging surgery with / without pelvine nodes

**Table 7:** Cox analysis (bivariate and multivariable) with disease-free survival as endpoint in patients with non-serous tumors.

Variable	Bivariate analysis		Multivariable analysis		P-value
	OR	95% CI	AOR	95% CI	
Age	1.01	0.97–1.05	1.01	0.97–1.05	0.723
Stage (I/II)	3.32	1.16–6.86	4.53	1.57–13.1	0.005
Type (I/II)	2.94	1.06–2.94	1.88	0.55–6.49	0.318
HRNPM positive	0.62	0.23–1.68	0.45	0.14–1.37	0.159
SLC1A5 positive	0.27	0.10–0.73	0.28	0.09–0.85	0.024

## Discussion

In the present study the relevance of HRNPM and SLC1A5 as prognostic factors for recurrent disease, survival and their impact on clinical and pathological features in a series of 123 patients with epithelial ovarian cancer in FIGO-stages I-II was evaluated. Furthermore, concomitant expression of the HRNPM and SLC1A5 proteins and p21 and p27, as cell cycle regulators, PTEN and PUMA, as regulators of apoptosis, and the angiogenic factor VEGF-R2 was explored. Because of the heterogeneity of the ovarian tumors studied, no single biologic parameter will give accurate prognostic information for all ovarian cancer patients. Therefore a combination of two or three separate factors may prove to give the best overall prognostic index [21,22].

HRNPM was found in the nucleus of the tumor cells and positivity for HRNPM was detected in 85 (61%) out of the 123 tumors in this study. According to the Human Protein Atlas [23] positivity (strong immuno-histochemical staining) for HRNPM was detected in 10 (83%) out of the 12 investigated tumors of ovarian cancer. HRNPM status was not associated with any clinical features, recurrent disease, or survival. In the Human Protein Atlas [23] results from immuno-histochemical staining is available for both normal ovarian tissue and epithelial ovarian cancer. Nuclear staining for HRNPM is also found in normal colon tissue, in both epithelial and stromal cells, and strong staining occurs in colon cancer. Several publications have investigated the importance of HRNPM in colorectal cancer and also its association with CEA [8,24–26]. For instance, Chen et al [25] has shown that the expression of HRNPM promotes progression of cancer development in colon tissue.

In the present study, positivity for HRNPM was more frequently detected in tumors with concomitant PUMA and p21 positivity, concomitant PUMA positivity and p21 negativity, or concomitant positivity for p21 and negativity for PUMA. Furthermore, negative staining for HRNPM was associated with concomitant negativity for PUMA and p21. Proteins of the HRNPM family lack enzymatic activity needed for DNA synthesis and phosphorylation. However, the HRNPM family of proteins acts as adaptor proteins and interacts with both DNA and RNA[27], and as some members of the HRNP gene family are capable of transforming normal cells into cancer cells, these genes are defined as oncogenes [5]. In the present study, concomitant positivity for HRNPM and PUMA and/or p21 could be explained by these properties, i.e. that presence of the HRNPM proteins in the tumor cells could activate induction of apoptosis and cell cycle arrest by PUMA and p21, respectively [16]. PUMA is normally expressed at a very low level in the tissues, but is rapidly induced in response of a wide range of stimuli. PUMA-mediated apoptosis acts as a safeguard against neoplastic transformation [28].

Rivera Vargas et al [29], has shown that the HRNPMs protein partner IMP-3 (insulin-like growth factor 2 mRNA-binding protein) is necessary for the posttranscriptional regulation of cyclines, which are proteins of importance for cell cycle regulation. The nuclear presence of IMP-3 is dependent of the presence of nuclear HRNPM, and accumulation of IMP-3 and HRNPM in the cytoplasm may inhibit proliferation, whereas nuclear accumulation, on the other hand, could stimulate synthesis of cyclin and proliferation.

HRNPM-positivity of tumors was more frequently detected in VEGF-R2 positive tumors. The role of VEGF-R2, by acting as receptors for VEGF-A, is regulation of tumor angiogenesis, which in turn is essential for solid tumor growth. Furthermore the key stages in the development of ovarian cancer are the passage of the carcinoma cells through the basic membrane and infiltration of adjacent tissues [30].

Positivity for SLC1A5 was detected in 92 (86%) out of the 121 available tumors in our study.

Huang et al [31] stained normal colorectal and colorectal cancer tissue from 92 patients for SLC1A5. Staining was positive in 83 of 90 (92%) colorectal cancer tissues, and their findings are in line with our results.

SLC1A5 positivity of tumors was found more frequently in tumors with p27, PTEN and PUMA positivity, respectively. Moreover, the SLC1A5 status was associated with PUMA and p27 status, where concomitant positivity for p27 and PUMA was the most striking finding. Lastly, SLC1A5-positive tumors were commonly also positive for VEGF-R2. Thus, it could be concluded, that the SLC1A5 protein has functions important for cell cycle regulation, tumor suppression, apoptosis and angiogenesis. SLC1A5 status was not associated with any clinical or pathological features in the whole series. However, in the subgroup of non-serous tumors, SLC1A5 negativity was associated with recurrent disease and worse disease-free survival. Thus, it was possible to identify a subgroup of patients, confined to 53 (43%) out of the 123 patients included in the study, with SLC1A5-positive non-serous tumors who had excellent 5-year disease-free survival, following adjustment for relevant confounders. Because disease-free survival was 92% at 5 years and 78% at 9 years, this subgroup of patients had favorable prognosis and could be considered as longtime survivors.

All the patients in this study had post-surgical chemotherapy, usually paclitaxel and carboplatin. The role of SLC transporters in chemo-resistance of human cancer is well described in the literature [31,32]. SLCs typically mediate uptake and chemo-sensitivity for hydrophilic drugs and one SLC can mediate uptake of different drugs. SLCs may also serve as chemo-resistance factors, reflecting functions other than a transporter-drug substrate relationship, as nutrient transporters may be up-regulated in tumor cells due to elevated energy needs. However, the SLC genes may be up- or down-regulated in the development of drug resistance [9]. As there was no differences in this study for SLC1A5 positivity between serous and non-serous tumors or the type of given post-surgical chemotherapy between those two main histological subgroups, it could be concluded, that the protective effect of SLC1A5 in non-serous carcinoma was not associated with drug resistance, but by some other biological property.

In summary the SLC1A5 status was an independent predictive factor for recurrent disease and a prognostic factor for disease-free survival in patients with non-serous epithelial ovarian cancer in FIGO-stages I-II after primary surgery and post-surgical single drug carboplatin or platinum/ taxane-based chemotherapy. As the staging procedure at the time of primary surgery according to the EORTC surgical staging was undertaken in only 34 (28%) of the 123 patients, it could not be guaranteed, that some patients had a FIGO-stage III disease. However, positive SLC1A5 status was still an independent predictive factor for recurrent disease together when staging surgery was incorporated in the multivariable model. Because of the heterogeneity of the tumors, no single biological parameter will give accurate prognostic information in all ovarian cancer patients and a combination of two or more independent factors may prove to yield the best overall prognostic index. A prognostic model for patients with the early stages (FIGO IA—IIC) non-serous ovarian carcinoma has been presented. An advantage of the prognostic model might be the fact that the FIGO stage is not included and therefore the problem concerning inaccurate surgical staging in patients with apparent early could be avoided [33].

## Conclusion

It seems that SLC1A5 protects from recurrent disease by means of its’ own biological properties in the subgroup of patients with non-serous tumors, as no difference in recurrent disease was found according to the type of postoperative chemotherapy given. Furthermore, the SLC1A5 status was significantly associated with recurrent disease in a subgroup of patients who all received the same type of chemotherapy (carboplatin and paclitaxel).

This is, to our knowledge, the first study indicating that SLC1A5 seems to be a prognostic factor for epithelial ovarian cancer. As the proteins HRNPM and SLC1A5 are related to the cell cycle regulators p21 or p27, the apoptosis regulators PTEN or PUMA, and the angiogenesis regulator VEGF-R2 it might be concluded, that both have a functional role in the pathogenesis of epithelial ovarian cancer.

## Supporting information

S1 Table Dataset corresponding to the presented findings. (XLSX)
